# A radiographic analysis of tooth morphology following the use of a novel cyclical force device in orthodontics

**DOI:** 10.1186/1746-160X-7-14

**Published:** 2011-08-09

**Authors:** Chung H Kau

**Affiliations:** 1 Department of Orthodontics, University of Alabama at Birmingham School of Dentistry, 1919 7th Avenue South, Room 305, Birmingham, AL 35294, USA

## Abstract

**Background:**

The purpose was to determine whether or not a novel device used in conjunction with orthodontic treatment produced root resorption shown on 3D images generated from a new cone beam computerized tomography.

**Methods:**

Subjects were actively recruited and those who received braces for the first time were invited to participate. Patients were assigned to receive a functioning device and used the devices for 20 min daily for a six month study period. CBCT images were taken of the dentition at the start of treatment and at the end of the study period.

**Results:**

14 subjects out of a possible 17 subjects completed using the device during the study period. The mean age of the subjects was 20.3 years. Measurements of all teeth present were made from the mesial buccal roots of the first molar on one side of the dental arch to the mesial buccal roots of the first molar on the opposing side of the same arch. These measurements were recorded as linear lengths in mm. A paired *t*-test was used to determine if significant differences occurred for root lengths at the end of treatment compared to the start of treatment for each of the individual tooth groups. No statistical differences were noted for root length changes above 0.5 mm and 1 mm.

**Conclusions:**

No statistically significant findings were noted for root length change at the end of treatment compared to the start of treatment when using this novel robotic device. No significant differences were noted between roots of anterior and posterior teeth. No clinically significant changes between root lengths were noted above 0.5 mm.

## Introduction

The clinical practice of orthodontics has been based on movement of teeth through alveolar bone using bio-mechanical methods within a safe, cellular environment. This technique involves the use of static mechanical forces to move teeth within the jawbone. The most common treatment approach is to correct malocclusion by providing these mechanical forces. This treatment has been used for approximately 100 years and involves a system of metal archwires and brackets, typically referred to as orthodontics. The basic system may be augmented with elastics, metal bands, head gear, retainers, and other ancillary devices as dictated by the specific and individualized treatment. These forces are static in that they are only adjusted at specific visits but then stay constant and do not change between visits.

Orthodontics works by applying steady pressure to the teeth (static forces), moving them gently and gradually into new positions according to the interaction of the archwire and bracket. Physiologically, this is possible because bone is constantly remodelling. When a tooth is pushed in a certain direction, the surrounding bone is remodelled. The direction of bending of the tooth is influenced by polarity created by the mechanical forces. When the tooth is under pressure and increased in convexity, the area is in an electropositive state. This state is associated with osteoclastic activity of bone resorption. When the tooth is under tension and increased in concavity, the area is in an electronegative state. This state is associated with osteoblastic activity of bone deposition [1].

Tooth movement may be considered an inflammatory process, and cytokines, such as interleukin-1 (IL-1), interleukin-6 (IL-6), and receptor activator of nuclear factor κB ligand (RANKL), are inflammatory or pro-inflammatory mediators remodelling the periodontal ligament (PDL) tissue [2]. The PDL is a connective tissue attaching the tooth to the alveolar bone. The tissue withstands the compressive forces during chewing while keeping the tooth in place. RANKL is reportedly essential to the osteoclast formation, function, and survival [3].

Some orthodontic researchers have suggested other methods to increase the rate of tooth movement by exploiting cellular processes. One such method is the use of corticotomies to accelerate tooth movement [4]. A recent article has even suggested that different types of surgical procedures create different effects in the surrounding bony areas facilitating a variable response to tooth movement [5].

In another study, it has been reported that low magnitude mechanical signals are "anabolic" to bone when applied at a high frequency. Long term use of this technique enhances bone stiffness and strength, and it also shows an increase in cancellous bone volume fraction, trabecular thickness, and trabecular number [6]. A light force produces significantly more tooth movement than heavier force application [7]. However, optimal force varies between patients along with the magnitude of the applied force affecting the rate of tooth movement [2]. Therefore, a device that transmits these forces may be an added benefit in orthodontic treatment.

However, use of such a device may pose a potential problem in root resorption. This condition is characterized by the loss of root cementum and dentin [8]. As a result, root resorption is a concern in orthodontic treatment and is thought to occur as a side-effect of cellular activity in the removal of the necrotic hyalinized tissue [2]. Root resorption is a precursor to the eruption of permanent teeth. However, root resorption of permanent teeth is an inflammation caused by varying factors, including injury to the root surface followed by dental trauma, surgical procedures, non-vital teeth bleaching, and mechanical procedures involving periodontal treatment [8].

The "gold standard" to measure root resorption is to sacrifice the tooth and surrounding alveolar bone and to histologically analyze the morphology. However, this type of analysis is not possible in a clinical setting. Therefore, a common method of evaluating root resorption is through conventional radiography. Some examples are panoramic radiography or peri-apical films. However, these models may be of limited use. A more accurate evaluation of root resorption can be achieved by analyzing cone beam computed tomography (CBCT) images. CBCT imaging has been moving toward providing greater amounts of information in regard to root morphology and periodontal structures [9].

This study represents the first human use of a novel cyclical device. The purpose of this study was to determine the effects a cyclical device may have on root lengths of teeth on 3D images generated from a new, computerized cone beam tomography device.

## Methods

Subjects who received braces for the first time were invited to participate, as long as they were within the first week of getting braces bonded. Patients were assigned to receive a functioning device and used the devices for 20 min daily for a six month study period. Study approval was given by the Institutional Review Board (IRB) at the University of Texas Health Science Center, Houston, TX, USA.

The inclusion criteria for subjects were as follows:

1. Permanent dentition

2. Class I malocclusion with crowding or spacing of ≥6 mm for mandibular incisors, lower number 1's through 3's

3. All patients will be candidates for canine retraction with bicuspid extraction

4. Predicted compliance with device use, as determined by the investigator orthodontist

5. Good oral hygiene, as determined by the investigator orthodontist

6. At least average intelligence, as determined by investigator orthodontist

The exclusion criteria for subjects were as follows:

1. Any medical or dental condition that in the opinion of the investigator could impact study results during the expected length of the study

2. Patient is currently using any investigational drug or any other investigational device

3. Patient plans to relocate or move within six months of enrollment

4. Allergic to acetaminophen (use of aspirin or non-steroidal anti-inflammatory drugs is excluded for patients while on the study)

5. Use of bisphosphonates, such as osteoporosis drugs, during the study

6. Pregnancy

### Novel device

**Figure 1:**
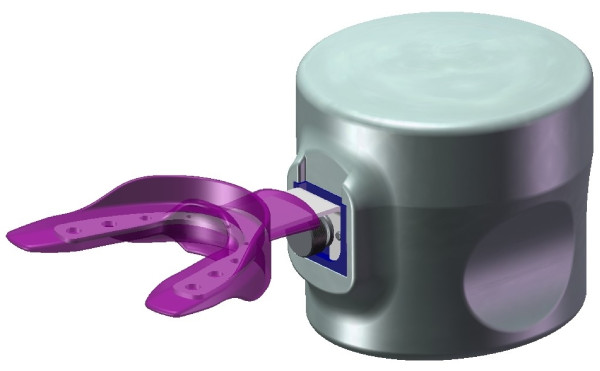
**An example of the AcceleDent Type 1 device**.

The novel device used for this study was the AcceleDent Type I (Figure 1). The device uses the application of cyclic forces to move teeth in bone faster through accelerated bone remodelling. The product is a removable orthodontic device, similar to a retainer, which attaches to the orthodontic archwire. In short, one part of the device is placed into the subject's mouth while the other end sits just outside the mouth and provides a small mechanical force to the teeth. The component outside the mouth shaped like a computer mouse and houses the mechanical, electrical, and energy components to activate the mechanical force from the post. The patient places and activates the device once daily for 20 min. The applied force (0.2-10 Newtons) is intended to be barely noticeable and should not be uncomfortable. Some researchers have theorized that the pulsing actually may decrease pain associated with standard orthodontic adjustments [10]. Importantly, AcceleDent is designed to work with all existing bracket technologies and is intended to complement rather than replace existing bracket technologies, such as braces.

### Imaging Device

The CBCT imaging device used for this study was the Sirona Galileos cone beam device. This system emits a radiation dose between 29 uSv to 54 uSv, as reported by the manufacturer. It has a scan time of 14 s and captures the maxilla-mandibular region in a 210° rotation within a radiation-detector configuration. The field of view is a spherical volume of 15 cm. The voxel size is between 0.15 mm to 0.30 mm, and the grayscale is 12 bit.

A reconstruction program calculated the entire image volume from the data of 200 individual exposures generated from a pulsed scan and required 3 min for image generation. Image manipulation was carried out using the manufacturer's software, Galaxis. To increase the accuracy of the assessment, all three planes (sagittal, axial, and coronal) were utilized.

### Parameters Measured

**Figure 2:**
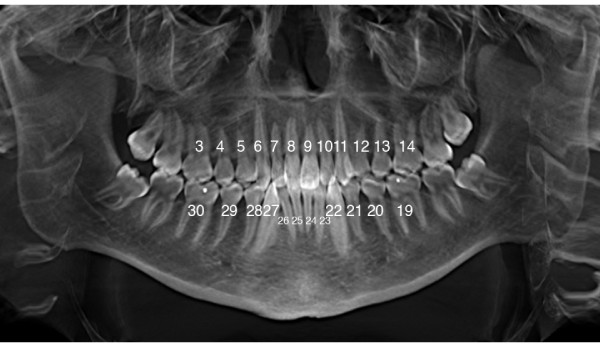
**Notation of Teeth**.

CBCT images were taken at two time frames; once at the start of treatment (T_1_) and again after six months of treatment (T_2_). Measurements of all teeth present were made from the mesial buccal roots of the first molar on one side of the dental arch to the mesial buccal roots of the first molar on the opposing side of the same arch (Figure 2). Linear root measurements were recorded in mm.

A further analysis was done to determine if groups of teeth reacted differently. For example, if the anterior teeth (canines and incisors) reacted differently to the posterior teeth (premolars and molars).

### Statistical Analysis

The mean of the root lengths were measured in mm and tested for normality. The differences between the pre-treatment and mid-treatment root lengths were analyzed by using *t *-tests (SPSS 16.0.1, Chicago, IL). Reductions in tooth root length were measured for significant differences at 0.5 mm and 1 mm.

## Results

The following results were obtained, and some of the results are presented in Tables 1 and 2.

**Table 1:** Table showing the mean changes in root lengths at T_2 _compared to T_1_

Teeth	N	Mean (mm)	Std Dev (mm)	Max (mm)	Min (mm)	P Sig at 0.5 mm	P Sig at 1 mm
3	14	-0.127	0.226	0.4	-0.58	NS	NS
4	14	-0.034	0.457	1.19	-0.75	NS	NS
5	11	-0.103	0.449	0.75	-0.85	NS	NS
6	14	-0.416	0.316	0.01	-0.92	NS	NS
7	14	-0.112	0.295	0.39	-0.66	NS	NS
8	14	-0.12	0.322	0.37	-0.746	NS	NS
9	14	-0.321	0.341	0.19	-1.07	NS	NS
10	14	-0.295	1.005	1.28	-3.39	NS	NS
11	14	0.176	1.453	5.1	-1.06	NS	NS
12	11	-0.222	0.234	0.19	-0.58	NS	NS
13	14	0.173	0.766	2.62	-0.47	NS	NS
14	14	-0.047	0.409	1.08	-0.57	NS	NS
19	13	-0.107	0.205	0.13	-0.5	NS	NS
20	12	0.271	0.804	2.54	-0.56	NS	NS
21	14	-0.176	0.562	1.06	-1.12	NS	NS
22	14	-0.06	0.48	1.14	-0.67	NS	NS
23	14	-0.081	0.163	0.29	-0.44	NS	NS
24	14	-0.284	0.44	0.29	-1.38	NS	NS
25	14	-0.336	0.442	0.18	-1.27	NS	NS
26	14	-0.302	0.613	0.64	-1.83	NS	NS
27	14	-0.079	0.686	2.12	-0.72	NS	NS
28	14	0.076	1.047	3.45	-0.69	NS	NS
29	13	-0.225	0.383	0.24	-1.27	NS	NS
30	13	-0.142	0.351	0.28	-0.74	NS	NS

The *p *values at 0.5 mm and 1 mm.

**Table 2:** Means of the differences in root lengths at T_2 _compared to T_1 _based on groupings of anterior and posterior teeth

Group	Mean	Std Dev	Std Err	p-value (< 0.05)
Anterior Teeth (Maxilla vs Mandible)	-0.01	0.65	0.10	0.09
Anterior Teeth versus Posterior Teeth(maxillia)	0.13	0.64	0.10	0.20
Anterior Teeth verus Posterior Teeth (mandible)	-0.14	0.57	0.09	0.13

T-test indicated no statistically significant differences in groupings.

### Subjects

17 subjects were recruited to participate in the study. 14 subjects completed using the device during the study period. 3 subjects declined to continue using the device for a variety of personal reasons and were not included in this study. The mean age of the subjects was 20.3 years. The oldest patient was 56.6 years, and the youngest was 12.1 years.

### Mean Root Lengths

Measurements of all teeth present were made from the mesial buccal roots of the first molar on one side of the dental arch to the mesial buccal roots of the first molar on the opposing side of the same arch. Measurements were recorded as linear lengths. The mean root lengths of the upper and lower teeth are presented in Table 1. The differences in mean root lengths ranged from -0.127 mm to -0.416 mm for both arches.

### Parameters measured

A paired *t*-test was used to determine if significant differences in root lengths occurred at the end of the study period compared to the start of treatment for each of the individual tooth groups. No statistical differences were noted for root length changes above 0.5 mm and 1 mm.

When groups of teeth were measured, the results showed no statistical differences in the amounts of root resorption between anterior and posterior teeth (Table 2).

## Discussion

This was the first study conducted in humans to determine the safety and efficacy of a novel device that uses medical robotics to assist in the rapid movement of teeth. State of the art 3D technology was employed to determine if the device caused problems to the roots of all teeth and whether root resorption occurred.

The device used in this study was the AcceleDent Type 1 device. This device provides a cyclical force in addition to the standard static force provided by orthodontics. Application of these cyclical forces induces accelerated remodelling of the bone in which teeth are embedded, thereby enabling them to move faster. In a series of rabbit experiments (N = 24), Mao showed that cyclical forces (2 Newtons at 0.2 Hz and 1 Hz for 20 min daily), provided in addition to the typical static forces (braces provided 24 hours per day), induced more cranial growth, sutural separation, and proliferation of osteoblast-like cells [11,12]. Histological evidence indicated wider separation of the premaxillomaxillary suture, frontonasal suture, and maxillopalatine suture associated with cyclic loading. In contrast, sutures associated with control and static loads were less separated. This evidence provides the scientific basis for using a cyclical device to decrease standard orthodontic treatment time. Additionally, a device that utilizes cyclic forces has been applied and approved for use in other areas of the body [13]. For example, the Juvent 1000 device maintains and/or enhances muscle strength, function, and postural stability.

Root resorption is a potential side effect of any orthodontic treatment. However, numerous factors have been acknowledged as potential precursors to enhanced root resorption. These factors include the duration of treatment, the magnitude of force application, the direction of tooth movement, and the method of force application (continuous versus intermittent) [8].

In this study, the AcceleDent device was used as an adjunct to routine treatment. The types of forces were cyclical in nature hence providing an almost pulsating nature. In addition, the device was used for only 20 min a day. The closest force characteristic that this device produced would be seen as an intermittent force, and these types of forces have been shown to allow cementum to heal and prevent further resorption [14-16].

Furthermore, there have been conflicting discussions of what is considered to be clinically significant root resorption. Some authors have stated that root resorptions in excess of 1/3 of root length were significant [17] whilst another study showed that resorptions at > 2 mm were considered present in up to 25% of cases [18]. This study showed that the changes in the root lengths at the end of the treatment compared to the start of treatment were not statistically significant at the 0.5 mm and 1 mm levels. This stringent amount of 0.5 mm was considered to be within clinically acceptable limits considering the study lasted for 6 months, and long term results were not available.

## Conclusions

The following are conclusions of the novel robotic device. No statistically significant changes were noted for root lengths at the end of treatment compared to the start of treatment. No significant differences were noted between roots of anterior and posterior teeth. No clinically significant changes between root lengths were noted above 0.5 mm.

## Competing interests

The author declares that they have no competing interests.
